# Surgical outcomes of aortic root replacement using the double-felt pledget technique in acute Stanford type A aortic dissection: A retrospective single-center study

**DOI:** 10.1097/MD.0000000000043583

**Published:** 2025-08-01

**Authors:** Saeki Watanabe, Hironobu Morimoto, Takashi Harada, Daisuke Futagami, Keijiro Katayama, Shogo Mukai

**Affiliations:** a Department of Cardiovascular Surgery, Fukuyama Cardiovascular Hospital, Fukuyama, Hiroshima, Japan.

**Keywords:** aortic dissection, aortic root replacement, double-felt pledget

## Abstract

Hemostasis at the aortic root during replacement surgery for acute Stanford type A aortic dissection (ATAAD) can be technically challenging due to tissue fragility, often leading to fatal bleeding. We adopted a double-felt pledget technique to improve hemostasis. This study aimed to evaluate the surgical outcomes of this technique. We retrospectively reviewed 231 emergency ATAAD surgeries performed at our hospital between January 2010 and August 2022. Of these, 24 patients underwent aortic root replacement using the double-felt pledget technique. Primary outcomes included postoperative bleeding, need for rethoracotomy, and in-hospital mortality. Long-term survival was assessed using Kaplan–Meier analysis. Among 24 patients (14 men, mean age 60 years), no in-hospital or 30-day mortality occurred. No patients required reoperation for bleeding. The average hospital stay was 28 days. During a mean follow-up of 3.7 years, only 1 patient died of an unknown cause, and 3 required further aortic interventions. The technique was particularly effective in reoperative cases and those with connective tissue disorders. The double-felt pledget technique achieved favorable hemostasis and surgical outcomes in aortic root replacement for ATAAD. This technique may contribute to improving operative safety in high-risk aortic root procedures.

## 1. Introduction

Aortic root replacement during emergency surgery for acute Stanford type A aortic dissection (ATAAD) is indicated in cases with root involvement, coronary malperfusion, or significant aortic regurgitation. Achieving hemostasis at the proximal anastomosis is particularly challenging due to the friable nature of dissected tissue. Inadequate hemostasis may necessitate rethoracotomy or result in perioperative mortality. Various techniques have been described to reinforce the root anastomosis, including the double sewing ring and flanged methods.^[1,2]^ Recently, other reinforcement strategies such as the modified sandwich technique and the turn-up anastomotic technique have been reported to achieve reliable hemostasis in fragile dissected tissue.^[3–5]^ These techniques aim to distribute suture tension evenly and minimize local stress on the dissected annulus.

We developed a method involving a double-felt pledget suture to address this challenge. This study investigates the mid-term outcomes of this technique and its role in promoting safe and effective aortic root replacement in ATAAD surgery.

## 2. Methods

### 2.1. Study design and ethical approval

This retrospective observational study included consecutive patients undergoing emergency surgery for ATAAD at Fukuyama Cardiovascular Hospital from January 2010 to August 2022. The study was approved by the institutional ethics committee of our hospital (approval no. 104), and informed consent for retrospective analysis was obtained from all patients or their families.

### 2.2. Patient selection

A total of 231 patients underwent emergency ATAAD surgery during the study period. Among them, 24 patients underwent aortic root replacement and were included in the present analysis. No exclusion criteria were applied.

### 2.3. Surgical technique

**Figure 1.**
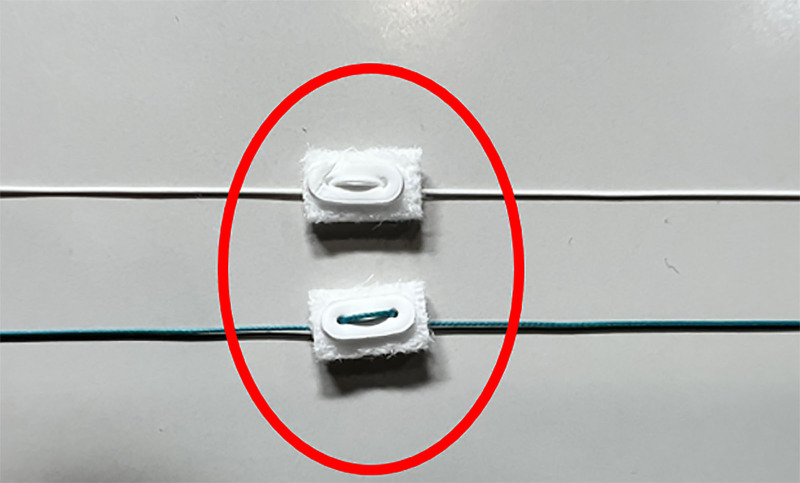
Double-felt pledget used at our hospital.

**Figure 2.**
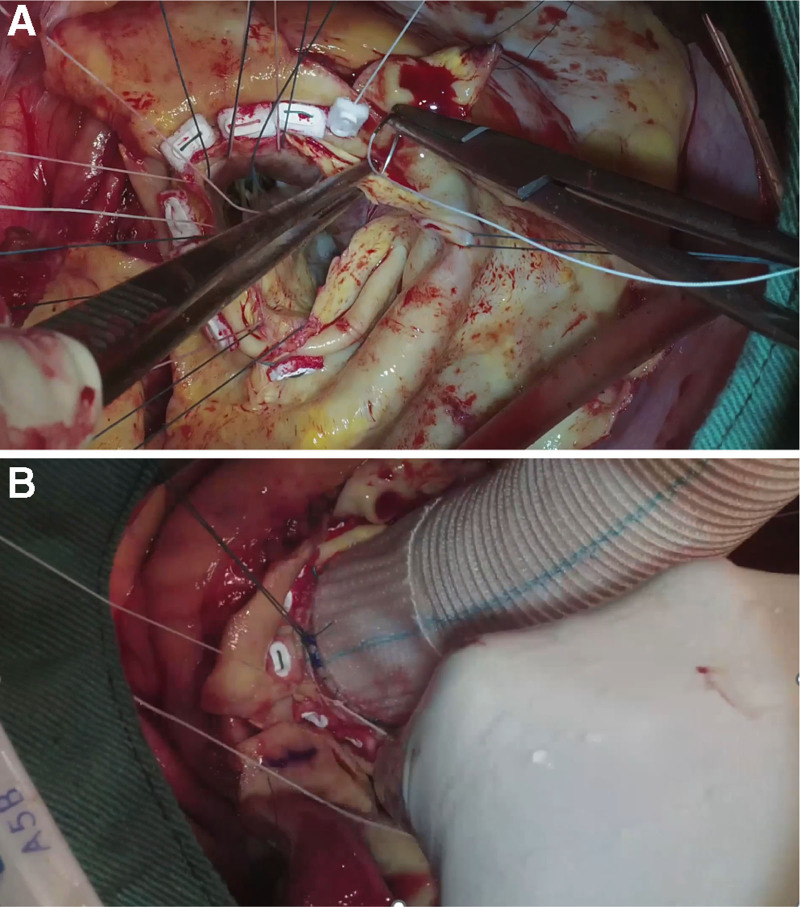
Image of the proximal anastomosis at the aortic root. (A) Suture the double-felt pledget in the intra-annular position of the aortic annulus. (B) Double-felt pledgets and the prosthetic graft are securely tied to ensure complete eversion.

Cardiopulmonary bypass was established via femoral or axillary cannulation. After cross-clamping and cardioplegic arrest, the root was excised and coronary buttons prepared. A composite graft was sutured to the annulus using a continuous everted suture technique with TEFDESSER II, a preconstructed double-felt pledget polyester suture (KONO SEISAKUSHO, Chiba, Japan) (Fig. 1). This technique allows even distribution of stress and improved hemostatic sealing between the prosthetic graft and dissected annular tissue (Fig. 2).

### 2.4. Outcome measures

Primary endpoints included operative mortality (defined as in-hospital or 30-day mortality), rethoracotomy for bleeding, and neurological complications. Long-term endpoints included overall survival and need for further aortic surgery. Kaplan–Meier analysis was used for survival estimation. Data were obtained from hospital records and outpatient follow-up.

## 3. Results

### 3.1. Patient characteristics

Of the 24 patients, 14 were male (58%) and the average age was 60 years (range: 42–73 years). Comorbid conditions included myocardial infarction (n = 3), cardiac tamponade (n = 2), severe aortic regurgitation (n = 5), prior cardiac surgery (n = 4), and Marfan syndrome (n = 2) (Table 1).

**Table 1:** Patient background.

Number (male)	24 (14)
Age	60.0 ± 11.4
Preoperative state Acute myocardial infarction Cardiac tamponade Severe AR Lower limb malperfusion	3 2 5 2
Operative indication Entry in the root Aortic root dilatation	19 5
Redo	4 (s/p AVR, ASD closure)
Marfan syndrome	2

AR = aortic valve regurgitation, ASD closure = atrial septal defect closure, s/p AVR = status post aortic valve replacement.

### 3.2. Surgical details

The Bentall procedure alone was performed in 12 patients, combined with arch procedures in the remainder (Table 2). Average cardiopulmonary bypass time was 306 minutes, cross-clamp time 230 minutes, and circulatory arrest time 42 minutes.

**Table 2:** Details of the surgical procedures.

Bentall	12
Bentall + TAR	4
Bentall + TAR + OSG	5
Bentall + PAR	1
Bentall + PAR + TAP	1
Aortic root repair	1

OSG = ostial graft, PAR = partial aortic root replacement, TAP = tricuspid valve annuloplasty, TAR = total aortic root replacement.

Coronary reconstruction methods included:

- Bilateral Carrel patch: 13 cases
- Bilateral interposition grafts: 5 cases
- Mixed techniques: 6 cases

No patient required rethoracotomy for bleeding, and no major neurologic complications (stroke, paraplegia) occurred. Two patients developed wound infections, and 1 required pacemaker implantation (Table 3).

**Table 3:** Postoperative outcome.

In-hospital death	0
30-d mortality	0
Hemorrhage	0
Stroke/cerebral hemorrhage	0
Paraplegia/paraparesis	0
Perioperative myocardial infarction	0
ECMO	1
Surgical site infection (femoral)	2
Sick sinus syndrome (pacemaker)	1
Hospitalization (d)	28.1 ± 10.8

ECMO = extracorporeal membrane oxygenation.

### 3.3. Follow-up

**Figure 3.**
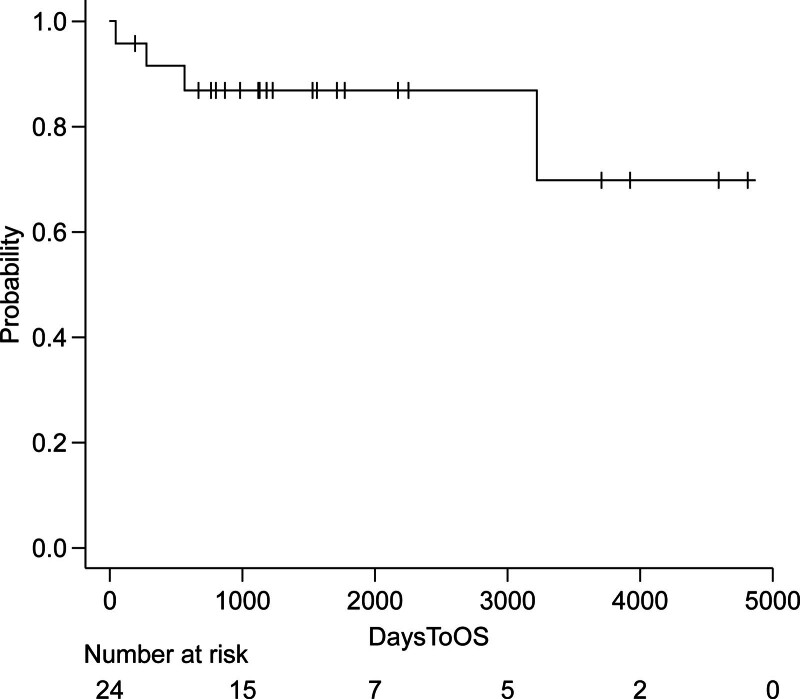
Survival rate. OS = overall survival.

Mean follow-up was 3.7 years (maximum 12 years). Three patients were lost to follow-up. One late death occurred due to an unknown cause. Three patients underwent further aortic repair (2 TEVAR, 1 open arch and descending replacement). The Kaplan–Meier survival estimate is shown in Figure 3.

## 4. Discussion

This study demonstrated favorable mid-term outcomes of aortic root replacement for ATAAD using a double-felt pledget technique. Importantly, we observed no in-hospital mortality, no reoperations for bleeding, and low rates of late adverse events.

With the double-felt pledgets, stress during ligation is not applied directly to the felt across the pledget, thereby allowing tissues weakened by aortic dissection to be gently ligated. Furthermore, the double-felt pledgets can be crimped to tissues more reliably than felt alone because of the double reinforcement with the felt and pledget, and a greater hemostatic effect can be achieved by everting the aortic root and the prosthetic graft during ligation. This technique almost certainly achieves hemostasis of the aortic root without the need for additional sutures at the aortic root. The double-felt pledget technique was found to have a great hemostatic effect, and even during root replacement surgery for aortic dissection, there was no need for a second row of sutures for the artificial blood vessel and the aortic root.

Compared with previously described reinforcement techniques such as the flanged technique or double sewing ring technique or modified button-Bentall operation, our double-felt pledget technique appears simple, reproducible, and effective in securing hemostasis in fragile dissected tissue.^[1,2,6]^ Similar to findings by Yang et al,^[7]^ our technique circumferentially distributes suture tension, reducing localized shear stress and preventing tear-through at the annulus.

The absence of bleeding-related reinterventions is especially meaningful in patients with connective tissue disorders or redo operations, where the risk of anastomotic failure is high. The technique also proved beneficial in high-risk, complex repairs involving arch and valve procedures.

## 5. Limitations

This study is limited by its retrospective, single-center design, and small sample size. The absence of a control group makes it difficult to isolate the independent effect of the double-felt pledget technique. Furthermore, the follow-up period, though reasonable, limits conclusions about long-term durability.

## 6. Conclusion

Aortic root replacement using the double-felt pledget technique in ATAAD surgery was associated with excellent hemostasis and low perioperative morbidity and mortality. This technique offers a valuable surgical option in high-risk root reconstruction.

## Acknowledgments

We would like to thank Editage (www.editage.jp) for English language editing.

## Author contributions

**Conceptualization:** Saeki Watanabe, Hironobu Morimoto.

**Data curation:** Saeki Watanabe, Takashi Harada, Daisuke Futagami.

**Formal analysis:** Saeki Watanabe, Keijiro Katayama, Shogo Mukai.

**Methodology:** Saeki Watanabe, Hironobu Morimoto.

**Writing – original draft:** Saeki Watanabe.
